# Prevalence of antibodies to *Leishmania infantum* and *Toxoplasma gondii* in horses from the north of Portugal

**DOI:** 10.1186/1756-3305-6-178

**Published:** 2013-06-17

**Authors:** Ana Patrícia Lopes, Susana Sousa, JP Dubey, Ana J Ribeiro, Ricardo Silvestre, Mário Cotovio, Henk DFH Schallig, Luís Cardoso, Anabela Cordeiro-da-Silva

**Affiliations:** 1 Department of Veterinary Sciences, School of Agrarian and Veterinary Sciences, University of Trás-os-Montes e Alto Douro (UTAD), Vila Real, Portugal; 2 Animal and Veterinary Research Centre, UTAD, Vila Real, Portugal; 3 Parasite Disease Group, Instituto de Biologia Molecular e Celular (IBMC), Universidade do Porto , Porto, Portugal; 4 Animal Parasitic Diseases Laboratory, Beltsville Agricultural Research Center, Agricultural Research Service, United States Department of Agriculture, Beltsville, MD, USA; 5 Veterinary Hospital, UTAD, Vila Real, Portugal; 6 Department of Parasitology, Koninklijk Instituut voor de Tropen (KIT), Royal Tropical Institute, Amsterdam, The Netherlands; 7 Departamento de Ciências Biológicas, Faculdade de Farmácia, UP, Porto, Portugal

**Keywords:** Antibodies, Horses, Leishmania infantum, Portugal, Toxoplasma gondii

## Abstract

**Background:**

*Leishmania infantum* and *Toxoplasma gondii* are protozoa with zoonotic and economic importance. Prevalences of antibodies to these agents were assessed in 173 horses from the north of Portugal.

**Findings:**

Antibodies to *L. infantum* were detected by the direct agglutination test (DAT); seven (4.0%) horses were seropositive with DAT titres of 200 (n = 5), 800 (n = 1) and ≥ 1600 (n = 1). Antibodies to *T. gondii* were assayed by the modified agglutination test (MAT); 23 (13.3%) horses were seropositive with MAT titres of 20 (n = 13), 40 (n = 5), 80 (n = 3) and ≥ 160 (n = 2). No statistical differences were found among equine categories of gender (female, male and gelding), age (1.5–6, 7–12 and 13–30 years), type of housing (indoors and mixed/outdoors), ability (recreation, farming and sports) and clinical status (apparently healthy and sick) for both agents.

**Conclusions:**

Horses are exposed to and may be infected with *L. infantum* and *T. gondii* in the north of Portugal.

## Findings

Protozoan parasites *Leishmania* spp. and *Toxoplasma gondii* are agents of zoonotic diseases with considerable importance worldwide [1,2]. Leishmaniosis caused by *Leishmania infantum* (syn. *Leishmania chagasi*) is endemic in the Mediterranean basin (including Portugal), the Americas, and Asia. Dogs are the main reservoir of the parasite, which is transmitted among animals and to humans by phlebotomine sand flies [3]. Infection with *L. infantum* in humans may cause severe visceral or mild cutaneous clinical outcomes, while canine leishmaniosis is a relatively frequent chronic viscerocutaneous and even fatal illness [4]. Cases of presumed autochthonous cutaneous leishmaniosis in horses have been sporadically reported from southern [5] and Central Europe [6].

Toxoplasmosis can be the cause of morbidity and mortality in congenitally infected children and imunocompromised people [1]. Felids are the only recognized definitive hosts of the parasite, while a large variety of mammals, including human beings and horses, serve as intermediate hosts. The consumption of improperly cooked infected horse meat can be a risk to human health, sometimes with serious consequences [7]. Recently, public attention has been drawn to adulteration of beef with horse meat in Europe.

The present study aimed at estimating the seroprevalences of *L. infantum* and *T. gondii* in horses from the north of Portugal, since no information is available on these zoonotic agents among the regional equine population.

Between November 2008 and July 2010, blood from 173 horses living in the north of Portugal was collected from a jugular vein into EDTA tubes and plasma stored at -25°C until use. Information on gender, age, type of housing and ability (Table 1) was obtained by filling in a questionnaire. The average age was 9.3 years (standard deviation: 5.8 years). Based on physical examination and clinical history, horses were considered as sick if they had fever, anorexia and/or intolerance to exercise. Those animals with no detectable clinical signs were regarded as apparently healthy (Table 1). This study was approved by the University of Trás-os-Montes e Alto Douro Veterinary Teaching Hospital ethical committee as complying with the Portuguese legislation for the protection of animals (Law no. 92/1995, 12 September).

**Table 1:** **Seropositivity among horses from the north of Portugal to *Leishmania infantum *and *Toxoplasma gondii *by gender, age group, type of housing, ability and clinical status**

	**Animals tested (n)**	**Relative distribution (%)**	***L. infantum*-seropositive**		***T. gondii*-seropositive**	
			**(n)**	**(%)**	**(n)**	**(%)**
Gender						
Female	78	45.1	3	3.8	11	14.1
Male	30	17.3	2	6.7	1	3.3
Gelding	65	37.6	2	3.1	11	16.9
Age (years)						
1.5–6	48	27.7	3	6.3	5	10.4
7–612	76	43.9	1	1.3	8	10.5
13–630	49	28.3	3	6.1	10	20.4
Housing						
Indoors	73	42.2	3	4.1	6	8.2
Mixed/outdoors	99	57.2	4	4.0	17	17.2
Not determined	1	0.6	0	0.0	0	0.0
Ability						
Recreation	109	63.0	7	6.4	14	12.8
Farming	30	17.3	0	0.0	4	13.3
Sports	30	17.3	0	0.0	5	16.7
Not determined	4	2.3	0	0.0	0	0.0
Clinical status						
Apparently healthy	148	85.5	5	3.4	21	14.2
Sick	14	8.1	2	14.3	2	14.3
Not determined	11	6.4	0	0.0	0	0.0
**Total**	**173**	**100**	**7**	**4.0**	**23**	**13.3**

The direct agglutination test (DAT) for titration of immunoglobulin G (IgG) antibodies specific to *Leishmania* followed the general procedures described by Schallig *et al*. [8], using a standard freeze-dried antigen at a concentration of 5 × 10^7^ promastigotes/ml (KIT Biomedical Research, Amsterdam, The Netherlands). Twofold dilution series ranging from 1:25 to 1:1600 were tested. Results obtained with the DAT are expressed as an antibody titre, i.e. the reciprocal of the highest dilution at which agglutination (large diffuse blue mats) is still clearly visible after 18 h incubation at room temperature. Plasma from a horse from the north of Portugal with cutaneous leishmaniosis (J. Elias and col., personal communication) and a DAT titre of 200 was used as positive control. Plasma from another horse (DAT titre < 25) that lived in a non-endemic region was used as negative control. To maximize specificity and sensitivity of the DAT, a cut-off titre of 200 was chosen for seropositivity.

The plasma samples were tested for IgG antibodies to *T. gondii* with a modified agglutination test (MAT) commercial kit (Toxo-Screen DA^®^, bioMérieux, Lyon, France) following the manufacturer’s instructions. Sera were analysed at the dilutions of 1:20, 1:40, 1:80 and 1:160. Positive and negative controls supplied with the kit were included in each plate. MAT positive results had visible agglutination (at least one half of the well’s diameter) after 5–18 h of incubation at room temperature. A cut-off titre of 20 (2 IU/ml in relation to a WHO international reference serum) was chosen to maximize both sensitivity and specificity of the test [9,10].

Chi-square or Fisher’s exact tests were used to compare percentages, including positivity levels among categories of the same independent variables (i.e. gender, age, type of housing, ability and clinical status) and total prevalences of antibodies to each one of the two agents. Exact binomial test established confidence intervals (CI) for the totals, with a 95% confidence level. Analyses were carried out using StatLib and SPSS 11.5 software for Windows, with a probability (*p*) value < 0.05 regarded as statistically significant [11].

Table 1 summarizes results. The seven (4.0%; 95% CI: 1.6–8.2) horses seropositive to *L. infantum* had titres of 200 (n = 5), 800 (n = 1) and ≥ 1600 (n = 1). The 166 horses seronegative to *L. infantum* had titres of < 25 (n = 156), 25 (n = 2) and 50 (n = 8). The 23 (13.3%; 95% CI: 8.6–19.3) horses seropositive to *T. gondii* had titres of 20 (n = 13), 40 (n = 5), 80 (n = 3) and ≥ 160 (n = 2). No horse was simultaneously seropositive to *L. infantum* and *T. gondii*.

This is the first epidemiological investigation on equine *Leishmania* and *T. gondii* infections in the north of Portugal, a region where both agents have previously been found to be endemic in other hosts species [12,13]. A higher level of exposure to *T. gondii* in comparison with *L. infantum* has been revealed in horses from northern Portugal, but gender, age, type of housing, ability and clinical status had no significant influence on the seropositivity to both parasites. The fact that no horse was simultaneously seropositive to *L. infantum* and *T. gondii* is a good indicator of the high specificity of the direct agglutination tests under use (i.e. the DAT and MAT).

Also by using the DAT, seroprevalences of *Leishmania* in dogs from the north of Portugal have been shown to range from less than 5% [14] to approximately 20% [12]. In domestic cats from this region prevalence found by means of the same serological test was 1.9% [15]. This is the first report of the DAT being used to investigate *Leishmania* infection in horses in Europe.

To the best of our knowledge, in Portugal only two clinical cases of equine cutaneous leishmaniosis have been reported: one from the north (J. Elias and col., personal communication) and the other one from the south-central region of Lisbon [16]. As previously observed in dogs, cats and humans, in areas of endemicity, the prevalence of subclinical *Leishmania* infection in horses is considerably higher than that of the disease [2,15,17].

In northern Portugal, out of 57 female *Phlebotomus ariasi* and *Phlebotomus perniciosus*, 38.6% were found to have fed on humans, 19.3% on dogs, 12.3% on chicken and 29.8% on other species, but no sand flies were detected with equine blood [18]. Nevertheless, in Spain *P. perniciosus* has been shown to feed on horses [19], a fact that does not exclude equines from taking part in the epidemiology of *L. infantum*.

Worldwide seroprevalences of *T. gondii* in horses were summarized by Dubey and Beattie [20] and Dubey [1]. In general, seroprevalence in horses is lower than in other livestock species and prevalences were higher in domestic than in wild horses [1]. The MAT titre that should be considered specific for the detection of antibodies to *T. gondii* in horses is unknown because only a few attempts have been made to isolate viable *T. gondii* from naturally exposed horses. Judging from results obtained with other species, a MAT titer of 20 is considered specific for the detection of antibodies to *T. gondii*. In the present study, 10 of the 23 seropositive horses had a titre of 40 or higher. Viable *T. gondii* was isolated from horses even with a very low titer of 4 in the Sabin Feldman dye test and horses experimentally infected with *T. gondii* oocysts do develop high MAT titres [9].

Horses are considered clinically resistant to toxoplasmosis and there are no confirmed cases of clinical toxoplasmosis in these animals [1]. The results of the present study indicate that horses in the north of Portugal are exposed to *T. gondii* and so their tissues might harbour parasitic cysts. The voluntary consumption of horse meat by people is not a common habit in Portugal, but the risk of *T. gondii* transmission from horses to humans should not be neglected due to the recent contamination of beef with horse meat noticed in Europe. Infections with *T. gondii* are common in food animals and humans in northern Portugal [13,21].

## Conclusions

In conclusion, horses are commonly exposed to and may be infected with *L. infantum* and *T. gondii* in areas where these zoonotic parasites are endemic, such as the north of Portugal. The degree of involvement of horses in the specific transmission of these agents to other hosts including humans is yet to be defined at the local level. However, in any case should equine infections with *L. infantum* or *T. gondii* be underestimated, respectively, as a potential cause of disease for horses themselves and as a risk to public health.

## Competing interests

The authors declare that they have no competing interests.

## Authors’ contributions

APL participated in serological testing and data analysis and drafted the manuscript; SS participated in serological testing and data analysis; JPD, RS, HDFHS and ACdS provided conceptual advice and reviewed the manuscript; AJR and MC collected samples and clinical data; LC participated in data analysis, helped to draft and revised the manuscript. All authors read and approved the final manuscript.
